# Oligonol, a Low-Molecular Weight Polyphenol Derived from Lychee, Alleviates Muscle Loss in Diabetes by Suppressing Atrogin-1 and MuRF1

**DOI:** 10.3390/nu9091040

**Published:** 2017-09-20

**Authors:** Hung-Wen Liu, Yen-Ju Chen, Yun-Ching Chang, Sue-Joan Chang

**Affiliations:** 1 Department of Life Sciences, National Cheng Kung University, No. 1, University Road, Tainan 701, Taiwan; hwliu@ntnu.edu.tw (H.-W.L.); ohaayan@gmail.com (Y.-J.C.); viyanchang@gmail.com (Y.-C.C.); 2 Department of Physical Education, National Taiwan Normal University, Taipei 106, Taiwan

**Keywords:** diabetes, Atrogin-1 and MuRF1, flavanol-rich lychee fruit extract, muscle loss, NF-κB

## Abstract

Stimulation of the ubiquitin-proteasome pathway—especially E3 ubiquitin ligases Atrogin-1 and MuRF1—is associated with muscle loss in diabetes. Elevated lipid metabolites impair myogenesis. Oligonol, a low molecular weight polyphenol derived from lychee, exhibited anti-diabetic and anti-obesity properties, suggesting it could be a proper supplement for attenuating muscle loss. Dietary (10 weeks) oligonol supplementation (20 or 200 mg/kg diet) on the skeletal muscle loss was investigated in diabetic *db/db* mice. Transcription factors NF-κB and FoxO3a involved in regulation of Atrogin-1 and MuRF1 were also investigated. Attenuation of muscle loss by oligonol (both doses) was associated with down-regulation of *Atrogin-1* and *MuRF1* gene expression. Oligonol supplementation decreased NF-κB expression in the nuclear fraction compared with *db/db* mice without oligonol supplement. Upregulation of sirtuin1 (SIRT1) expression prevented FoxO3a nuclear localization in *db/db* mice supplemented with oligonol. Marked increases in AMPKα activity and *Ppara* mRNA expression leading to lower lipid accumulation by oligonol provided additional benefits for attenuating muscle loss. Oligonol limited palmitate-induced senescent phenotype and cell cycle arrest and suppressed *Atrogin-1* and *MuRF1* mRNA expression in palmitate-treated C2C12 muscle cells, thus contributing to improving the impaired myotube formation. In conclusion, oligonol-mediated downregulation of *Atrogin-1* and *MuRF1* gene expression alleviates muscle loss and improves the impaired myotube formation, indicating that oligonol supplementation may be useful for the attenuation of myotube loss.

## 1. Introduction

Muscle atrophy is a decrease in the mass of skeletal muscle, and is observed in metabolic disorders such as diabetes and obesity [1,2]. Activation of ubiquitin-proteasome proteolytic pathways and inflammatory responses has been implicated in the pathogenesis of muscle atrophy [2,3,4]. Muscle atrophy f-box (MAFbx)/atrogin-1 and muscle RING-finger protein 1 (MuRF1)—two muscle-specific E3 ubiquitin ligases—are responsible for ubiquitin-mediated protein degradation [5]. Transcriptional activities of MuRF1 and Atrogin-1 are regulated by transcriptional factors nuclear factor-κB (NF-κB) and forkhead box O3 (FoxO3a) [3,6], suggesting that inhibition of NF-κB and/or FoxO3a pathways is a potential target to protect muscle against atrophy.

Myogenesis also plays a role in the maintenance of skeletal muscle mass in addition to protein degradation [4]. Recent reports indicated that the regenerative capacities of skeletal muscle were vulnerable to certain pathological conditions [7,8]. Aberrant cell cycle regulation and a senescent phenotype induced by ceramide lead to reduced myoblast proliferation [9]. Palmitate (PA)-induced insulin resistance disrupts the regulation of myotube formation [10]. These results point out the negative impacts of intramuscular lipid accumulation on myogenesis.

Biological functions of flavonoids in anti-inflammation, anti-diabetes, and anti-obesity have been reported [11,12]. The flavanol-rich lychee extract oligonol, consisting of catechins, procyanidins, and other phenolic compounds [13], has been shown to reduce abdominal fat in healthy subjects [14] and was shown to prevent high-fat diet-induced obesity in our previous study [12], indicating that oligonol may ameliorate metabolic syndrome. Oligonol inhibited NF-κB activation under different pathological conditions, including diabetes-induced kidney damage [15], diabetes, CCL4-induced liver injury [16,17], and dextran sulfate sodium-induced colitis [18] in vivo. However, the involvement of oligonol in the regulation of muscle loss has not been elucidated. The primary goal of the present study is to explore the role of oligonol in the inhibition of MuRF1 and Atrogin-1 via NF-κB and/or FoxO3a pathways to protect muscle against atrophy in obese diabetic *db/db* mice. The senescent phenotype, cell cycle, and myotube formation influenced by oligonol were investigated in palmitate-treated C2C12 muscle cells.

## 2. Materials and Methods

### 2.1. Materials

Oligonol (Amino Up Chemical Co., Sapporo, Japan) was derived from lychee fruit extract and green tea extract, and the major active components are flavanol monomers (catechin, epicatechin, epicatechin gallate, and epigallocatechin gallate (EGCG), 13–18%), dimers (procyanidin A1, A2, B1, and B2 and epicatechin-EGCG, 14–18%), trimers (epicatechin-procyanidin A2, 2–6%), and other phenolic compounds (other trimers, tetramers, oligomers, and polymers, ≥45%) [13]. For Western blotting, primary antibodies: sirtuin1 (SIRT1) and histone 3 were purchased from Abcam (Cambridge, MA, USA). Akt, phospho-Akt (Thr308), FoxO3a, phospho-FoxO3a (Ser253), AMP-activated protein kinase α (AMPKα), phospho-AMPKα (Thr172) were purchased from Cell Signaling (Danvers, MA, USA). GAPDH was purchased from GeneTex Inc. (Hsinchu City, Taiwan). NF-κB was purchased from Millipore (Billerica, MA, USA). Goat anti-rabbit and sheep anti-mouse horseradish peroxidase (HRP) conjugated secondary antibodies were purchased from Bio-Rad (Hercules, CA, USA) and GE Healthcare Life Sciences (Pittsburgh, PA, USA), respectively.

### 2.2. Experimental Animals

Animal experiments were approved by the Institutional Animal Care and Use Committee of National Cheng Kung University (Approval Number: 104124). Four-week-old male diabetic C57BLKS/J (*db/db*) mice (*n* = 10) and their age-matched corresponding control (*m/m*, *n* = 10) mice were purchased from the National Laboratory Animal Center (Taiwan). Two to three mice were caged in an air-conditioned animal facility at 20 ± 2 °C, 50 ± 5% humidity, and 12 h light/dark cycle with free access to water and normal chow diet (LabDiet 5058, St. Louis, MO, USA). Body weight was measured weekly. At the age of 8 weeks, *db/db* mice were divided into three groups: *db/db* mice (*n* = 10) were fed chow diet; *db/db* mice administered oligonol (20 mg or 200 mg oligonol/kg chow diet; *n* = 10 in each group) for 10 weeks. Several *db/db* mice died during the experimental period of oligonol treatment. At the end of treatment, the numbers of animals in *db/db*, oligonol 20, or oligonol 200 groups were 7, 8, and 7, respectively. The causes of early death in these animals may be due to ketonuria, hematuria, and gastrointestinal bleeding [19]. Animals were anesthetized by an intraperitoneal injection of pentobarbital (90 mg/kg body weight) followed by decapitation between 2 to 3 pm. Tibialis anterior muscles were fixed with 4% paraformaldehyde and gastrocnemius muscles were stored at −80 °C for further analysis.

### 2.3. Skeletal Muscle Histology

Embedded tibialis anterior muscle blocks from four groups were cut into 5 μm sections and stained with hematoxylin-eosin. Images were observed under a microscope and captured with a digital camera (Olympus, Tokyo, Japan). Tibialis anterior muscle was embedded in optimal cutting temperature compound (VWR, Radnor, PA, USA) and was cut into 20 μm sections. The sections were stained with 0.4% oil red O (ORO) solution (Sigma, St. Louis, MO, USA). Quantification of neutral lipids by ORO analysis was done using ImageJ (National Institutes of Health, Bethesda, MA, USA).

### 2.4. Western Blot Analyses

The protein levels were assessed by Western blot analysis as reported in the previous study [20]. Nuclear fraction was prepared by using nuclear extraction kit according to the manufacturer’s instructions (Abcam, Cambridge, MA, USA). Homogenates of gastrocnemius muscles were separated by sodium dodecyl sulfate polyacrylamide gel electrophoresis (SDS-PAGE), transferred to nitrocellulose membrane, and incubated with appropriate antibodies. Protein bands were visualized using Chemiluminescence kit (Millipore, Billerica, MA, USA) and quantified by using the LAS-4000 mini biomolecular imager (GE HealthCare Life Sciences, Pittsburgh, PA, USA).

### 2.5. RNA Extraction and Real-Time PCR

Total RNA was extracted from gastrocnemius muscle and C2C12 myotubes by using Trizol/chloroform procedure (Gibco BRL, Rockville, MD, USA) and quantified by the NanoDrop meter. Total RNA (1 μg) was reverse transcribed into cDNAs by using the iScript cDNA synthesis kit (Bio-Rad, Hercules, CA, USA). More detailed information on real-time PCR can be found in our previous study [20]. Primer sequences are shown in Table 1.

### 2.6. C2C12 Muscle Cell Culture and Differentiation

Mouse myoblast cell (C2C12) was obtained from the Food Industry Research and Development Institute (FIRDI, Hsinchu, Taiwan). C2C12 myoblasts were maintained in low glucose (1 g/L) Dulbecco’s modified Eagle medium (DMEM) supplemented with 10% fetal bovine serum (GE HealthCare Life Sciences, Pittsburgh, PA, USA). Twenty-four hours after seeding, myoblasts were pretreated with oligonol (10, 25, 50 μg/mL) for 16 h and incubated with palmitate (250 μM) for 8 h, followed by cell senescence and cell cycle analysis. After reaching 100% confluence, cells were incubated in low-glucose DMEM supplemented with 2% horse serum to induce differentiation. After 5-day differentiation, differentiated myotubes were serum starvation alone, or serum starvation supplemented with oligonol (50 μg/mL) for 3 h, incubated with palmitate (250 μM) for another 16 h, followed by real-time PCR analysis.

### 2.7. Cell Senescence

Following the treatment, cells were washed, fixed, stained with β-galactosidase staining kit using X-gal as the substrate (BioVision, Milpitas, CA, USA), and then incubated for 72 h at 37 °C. Images were observed under phase contrast microscopy and captured with a digital camera (Nikon, Tokyo, Japan).

### 2.8. Cell Cycle Analysis

Following the treatment, cells were trypsinized and then fixed with ice cold 100% ethanol. Following a cold phosphate-buffered saline (PBS) wash, cells were resuspended in 1 mL PBS containing 0.5% trition X-100 (Sigma-Aldrich, St. Louis, MO, USA) and RNase (Thermo Fisher Scientific, Waltham, MA, USA), incubated for 30 min at 37 °C, and then stained with propidium iodide (Sigma-Aldrich, St. Louis, MO, USA) for another 30 min. Cell cycle analysis was done using a flow cytometer (FACS Canto II, BD Biosciences, San Jose, CA, USA).

### 2.9. Myotube Formation and Crystal Violet Staining

Myoblasts receiving any other treatment during the proliferating phase (72 h after seeding) continued incubation for 5 days in low-glucose DMEM supplemented with 2% horse serum, followed by crystal violet staining. Myotubes were fixed with 4% paraformaldehyde (Sigma-Aldrich, St. Louis, MO, USA) and then stained with 1% crystal violet solution (Sigma-Aldrich, St. Louis, MO, USA). Images were observed under phase contrast microscopy and captured with a digital camera (Nikon, Tokyo, Japan). For measuring myotube formation, the number, diameter, and length of myotubes were analyzed using ImageJ (National Institutes of Health, Bethesda, MD, USA) as previously described [21].

### 2.10. Statistical Analysis

Data are expressed as mean ± standard error of the mean (SEM). The statistical significance of the differences among the groups (*p* < 0.05) was determined by one-way analysis of variance (ANOVA) and following post hoc assessment by Student–Newman–Keuls method correction for multiple comparisons (SigmaPlot 12.0, San Jose, CA, USA). Different lowercase letters indicate significant differences among groups.

## 3. Results

### 3.1. Effects of Oligonol on Body Weight, Muscle Fiber Sizes, Distribution of Fiber Sizes

**Figure 1:**
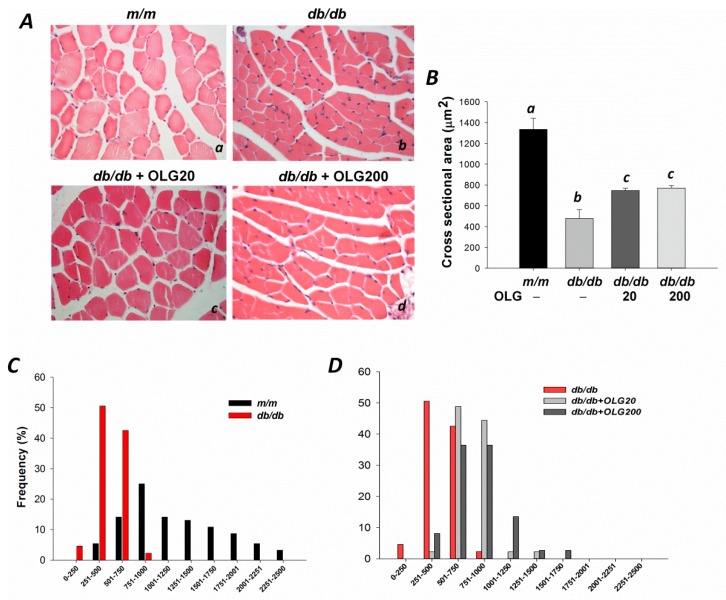
The cross-sectional area of tibialis anterior muscle in *m/m*, *db/db* mice, and *db/db* mice with oligonol (OLG) supplementation. (**Aa**–**d**) Myofibers were stained with hematoxylin-eosin (400×); (**B**) Mean cross-sectional area of tibialis anterior muscle; (**C**) The distribution of myofiber sizes in tibialis anterior muscles from *m/m* and *db/db* mice; and (**D**) *db/db* mice with or without oligonol (OLG) supplement.

At 18 weeks of age, body weights of *db/db* mice supplemented with or without oligonol were significantly higher than age-matched *m/m* mice (26.3 g ± 0.4 g vs. 45.8 g ± 1.2 g, 45.0 g ± 1.3 g, 46.1 g ± 1.3 g, *p* < 0.05). The average cross-sectional area of tibialis anterior muscle in *db/db* mice without oligonol supplementation was significantly smaller than that in age-matched *m/m* mice (Figure 1A(a,b),B). The average cross-sectional area was significantly increased by 1.5- and 1.6-fold after oligonol supplementation (20 and 200 mg/kg diet) compared with that in *db/db* mice without oligonol supplement (Figure 1A(b–d),B). The distribution of fiber sizes in *db/db* mice was observed to be shifted toward smaller fibers (0–1000 μm^2^) (Figure 1C). Oligonol supplementation in *db/db* mice prevented this shift, with fiber sizes ranging from 251 to 1750 μm^2^ (Figure 1D).

### 3.2. Effects of Oligonol on Atrogin-1 and MuRF1 mRNA Expression, NF-κB Activation, and FoxO3a Activation

**Figure 2:**
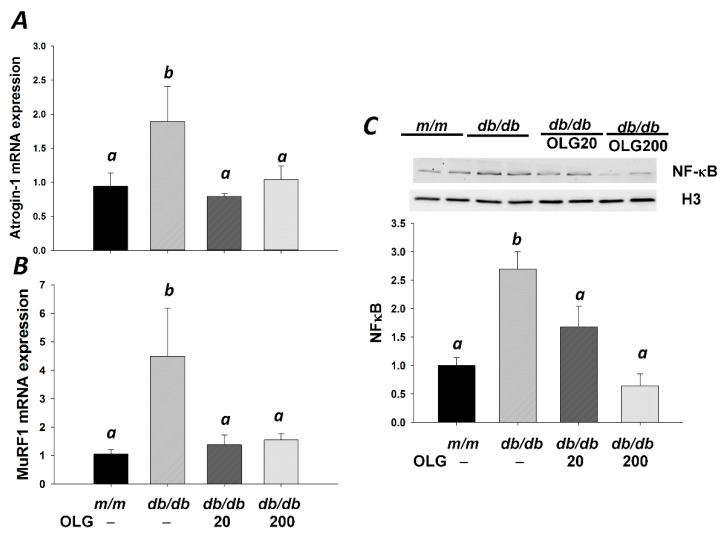
Gene expression was analyzed using real-time PCR analysis in gastrocnemius muscle from *m/m* and *db/db* mice with or without oligonol (OLG) supplementation. *Atrogin-1* (**A**) and *MuRF1* (**B**) mRNA expression is expressed as mean ratio to control after normalization with 18S mRNA levels. Fold differences were calculated using the ΔΔCt method. Values presented are mean ± SEM (*n* = 5–6/group). Protein expression was analyzed using Western blotting with anti-NF-κB and -Histone H3 antibodies in gastrocnemius muscle (**C**, *n* = 4–5/group). Significance (*p* < 0.05) among groups is denoted by different letters.

**Figure 3:**
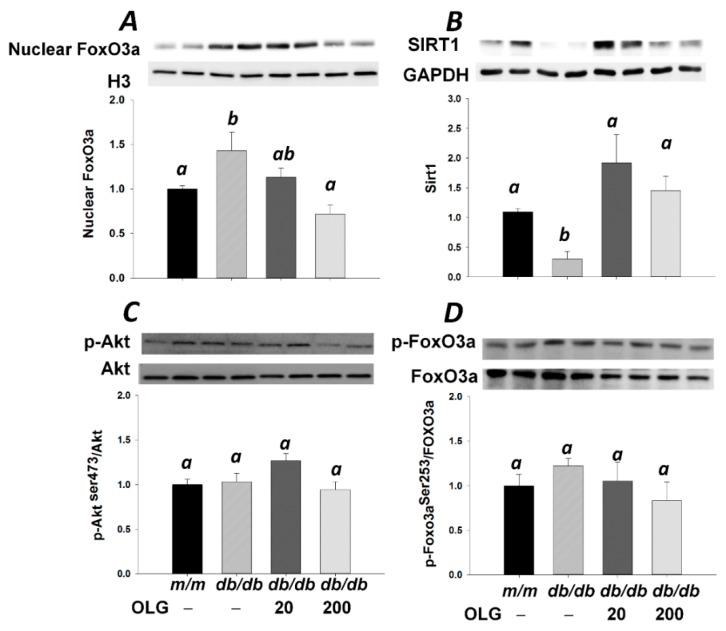
Representative blots of nuclear FoxO3a (**A**), SIRT1 (**B**), phospho-Akt (Thr473), Akt (**C**), phospho-FoxO3a (Ser253), and total FoxO3a (**D**) in *m/m* and *db/db* mice with or without oligonol (OLG) supplement are shown (*n* = 5–6/group). Significance (*p* < 0.05) among groups is denoted by different letters.

Muscle atrophy-related genes *Atrogin-1* and *MuRF1*—two muscle-specific E3 ubiquitin ligases—were upregulated in *db/db* mice without oligonol supplement (Figure 2A,B). *Atrogin-1* and *MuRF1* mRNA expressions were downregulated upon oligonol supplementation (Figure 2A,B), thus contributing to alleviating muscle loss in diabetic mice (Figure 1A,B). Atrogin-1 and MuRF1 expressions were regulated by nuclear factor-κB (NF-κB). Western blot analysis revealed a marked increase in NF-κB expression in the nuclear fraction of *db/db* mice compared with *m/m* mice (Figure 2C). Oligonol supplementation reduced NF-κB expression in the nuclear fraction compared with *db/db* mice without oligonol supplement (Figure 2C). FoxO3a is an important transcriptional factor for regulation of *Atrogin-1* and *MuRF1* gene expression. Similar to NF-κB nuclear localization, increased FoxO3a expression in the nuclear fraction was observed in *db/db* mice (Figure 3A). The nuclear FoxO3a expression was downregulated in *db/db* mice upon supplementation with high-dose oligonol (200 mg/kg diet). SIRT1 deacetylates and represses transactivation of FoxO3a. Oligonol supplementation elevated SIRT1 expression which was partially associated with blocked nuclear translocation of FoxO3a (Figure 3A,B). There was no difference in phosphorylation of Akt and FoxO3a among four groups (Figure 3C,D).

### 3.3. Effects of Oligonol on Lipid Accumulation

**Figure 4:**
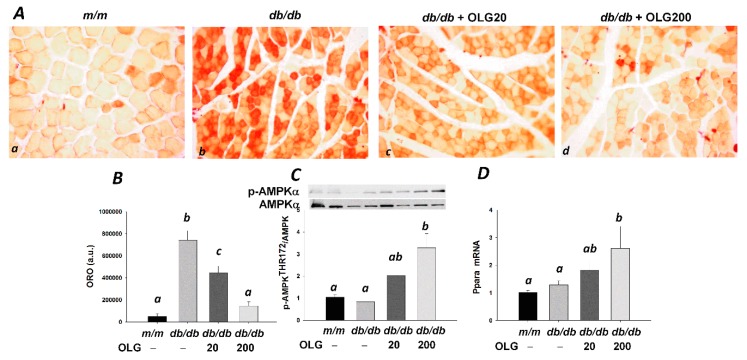
Oil red O (ORO)-stained tibialis anterior muscle sections (**Aa**–**d**) in *m/m* and *db/db* mice with or without oligonol (OLG) supplementation (200×); (**B**) Quantification of neutral lipids stained with ORO solution in tibialis anterior muscle (*n* = 3/group); (**C**) Representative blots of AMPKα and phospho-AMPKα (Thr172) are shown (*n* = 5–6/group); (**D**) *Ppara* mRNA expression level is expressed as mean ratio to control after normalization with 18S mRNA levels (*n* = 5–6/group). Fold differences were calculated using the ΔΔCt method. Values presented are mean ± SEM. Significance (*p* < 0.05) among groups is denoted by different letters.

Excessive lipid accumulation is associated with muscle loss. Lipid droplets were detected in skeletal muscle by oil red O (Figure 4A(a–d)). Increased intramuscular lipid accumulation in *db/db* mice skeletal muscle was ameliorated by oligonol, as evidenced by less lipid droplets (Figure 4A(b–d),B). Increased AMPKα activity and peroxisome proliferator-activated receptor α (*Ppara*) mRNA expression were observed in *db/db* mice supplemented with oligonol, in a dose-dependent manner (Figure 3C,D). Decreased intramuscular lipid accumulation was associated with activation of AMPKα/PPARα-mediated fatty acid oxidation.

### 3.4. Effects of Oligonol on Senescence, Cell Cycle, and Myotubes Formation in C2C12 Muscle Cells Treated with Palmitate

**Figure 5:**
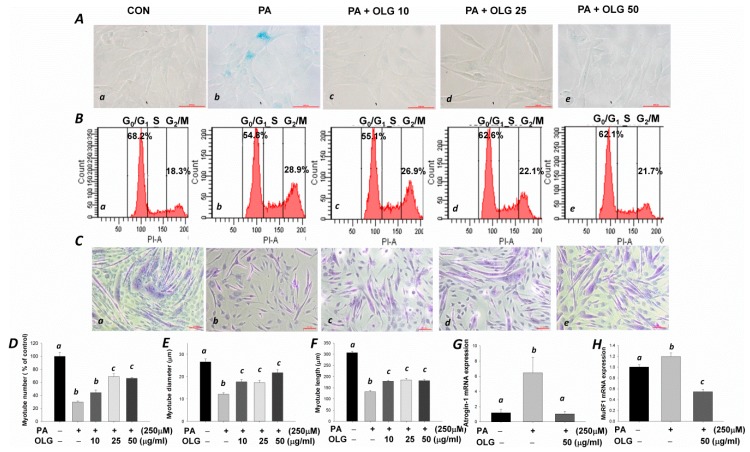
C2C12 myoblasts were pretreated with or without oligonol (10, 25, 50 μg/mL) for 16 h, followed by incubation with or without palmitate (PA, 250 μM) for 8 h. (**Aa**–**e**) Senescence-associated β-galactosidase positive cells exhibited blue-green color (400×); (**Ba**–**e**) The relative percentage of myoblasts at G0/G1, S, and G2/M phases of the cell cycle; (**Ca**–**e**) Myoblasts received different treatments during the proliferative phase were differentiated into myotubes; After 5 days of differentiation, crystal violet staining was used to examine myotube formation (100×); (**D**–**F**) Quantification of myotube number, diameter, and length, respectively; (**G**,**H**) *Atrogin-1 and MuRF1* mRNA levels in differentiated C2C12 myotubes treated with PA and/or oligonol. Fold differences were calculated using the ΔΔCt method. Values presented are mean ± SEM (*n* = 3/group).

The senescence β-galactosidase staining with strong blue-green color was observed in the palmitate-treated myoblasts (Figure 5A(b)). A significantly increased proportion of cells in G2-phase was observed in PA compared with control (CON) group (28.9 vs. 18.3%, Figure 5B(a,b)). The proportion of cells in G2-phase was decreased with the increasing doses of oligonol (Figure 5B(c–e)). Crystal violet staining and quantitative analysis of myotube formation showed fewer myotubes in palmitate-treated group (Figure 5C(b),D). These myotubes were smaller and shorter in diameter and length, respectively (Figure 5E,F), suggesting that palmitate-induced senescent phenotype and aberration in cell cycle regulation during the proliferative phase resulted in retarded myotube formation. Weak β-galactosidase positive signals (Figure 5A(c–e)) in accordance with decreased proportion of cells in G2-phase (26.9, 22.1, and 21.7%) in a dose-dependent manner were observed in oligonol-treated myoblasts (Figure 5B(c–e)). Oligonol significantly improved the impaired myotube formation compared with the palmitate-treated group (Figure 5C(c–e),D–F). Marked decreases in *Atrogin-1* and *MuRF1* mRNA levels were observed in oligonol-treated differentiated C2C12 muscle cells compared with palmitate-treated group (Figure 5G,H).

## 4. Discussion

Muscle loss is associated with increased muscle protein degradation in diabetes [22]. This catabolic condition is associated with NF-κB activation and stimulation of the ubiquitin-proteasome proteolytic pathway [3,4]. In the present study, we first demonstrated that a novel dietary supplement—oligonol—successfully inhibited muscle atrophy-related ubiquitin ligases atrogin-1 and MuRF1.

Atrogin-1 and MuRF1—two muscle-specific E3 ubiquitin ligases—are key regulators of ubiquitin-mediated protein degradation in skeletal muscle [22]. Activation of NF-κB leading to upregulated gene expression of *Atrogin-1* and *MuRF1* (atrophy-related genes) suggests that repressing NF-κB activation is a target for the prevention of muscle atrophy. In the present study, inhibition of NF-κB nuclear localization and downregulation of *Atrogin-1* and *MuRF1* gene expression were in accordance with greater size of myofibers in *db/db* mice upon oligonol supplementation. Inhibition of NF-κB by oligonol has been shown in our previous study [15] and other pathological conditions [16,17,23]. The anti-inflammatory property of oligonol could be derived from its major constituents, catechins and procyanidins. In line with our observations, cocoa flavanols containing flavan-3-ols and procyanidins exhibited anti-inflammatory property through inhibition of NF-κB activation [24]. Supplementation of epicatechin-rich cocoa reversed muscular dystrophy in type 2 diabetic patients [25]. Animal experiments and the clinical trial indicate that NF-κB is an important target for the treatment for muscle atrophy.

In the present study, upregulation of atrophy-related ubiquitin ligases atrogin-1 and MuRF1 was associated with FoxO3a nuclear localization in *db/db* mice. Phosphorylation of Akt and its downstream target FoxO3a was not observed in the present study, suggesting that FoxO3a dephosphorylation leading to nuclear translocation is not a major mechanism in this case [6]. SIRT1 deacetylates and represses the activity of FoxO3a [26], indicating that SIRT1-mediated deacetylation of FoxO3a could be the underlying mechanism responsible for FoxO3a nuclear localization. Dramatically decreased SIRT1 expression in accordance with marked increases in *Atrogin-1* and *MuRF1* gene expressions leading to muscle atrophy was observed after food deprivation (48 h) [27]. On the contrary, overexpression of SIRT1 reduced muscle atrophy by blocking FoxO3a activation [27]. In the present study, oligonol elevated SIRT1 expression and blocked FoxO3a nuclear localization. Recently, upregulation of SIRT1 expression by oligonol has been demonstrated in our [12] and other experimental models [28]. Based on these results, we concluded that oligonol downregulated *Atrogin-1* and *MuRF1* expressions by suppressing the nuclear localization of transcription factors NF-κB and FoxO3a.

NF-κB activation is also triggered by the accumulation of intramyocellular lipids [29]. In the present study, inhibition of NF-κB activation driven by lower cellular lipid contents was observed in *db/db* mice with oligonol supplement. Increases in AMPKα activity and *Ppara* mRNA expression were involved in fatty acid oxidation and subsequently reduced intramyocellular lipid contents in *db/db* mice with oligonol supplement. The efficacy of oligonol on lipid catabolism has been reported by in vitro and in vivo studies [14,17,30,31,32]. Similar to oligonol-mediated lipid metabolism, cocoa and its derived products promoted lipid catabolism through stimulation of AMPKα and PPARα pathways [33,34]. Therefore, oligonol suppresses lipid accumulation in skeletal muscle by promoting fatty acid oxidation, which provides additional benefits for the prevention of NF-κB-mediated muscle loss.

The restoration of muscle mass after disease, injury, or surgery relies on muscle regeneration. In the present study, impaired myogenesis induced by palmitate was directly related to the senescent phenotype, cell cycle arrest, and impaired myotube formation. Inhibition of myoblast proliferation by palmitate was associated with marked decreases in cyclin A and cyclin D1 levels and increase in p21 levels, thus leading to aberrant cell cycle regulation [35]. Our study showing that oligonol eliminates senescent phenotype and normalizes aberrant cell cycle regulation was consistent with the study using metformin to limit ceramide-induced senescence in C2C12 myoblasts [9]. In the same study, pretreatment of metformin attenuated ceramide-induced up-regulation of p21 proteins [9]. In line with our in vivo results, inhibition of *Atrogin-1* and *MuRF1* mRNA expressions by oligonol in differentiated muscle cells was able to improve the impaired myotube formation. Pharmaceutical agents or food supplements targeting the ubiquitin-proteasome pathway may be useful for the prevention of myotube loss.

## 5. Conclusions

Inhibition of atrogin-1 and MuRF1 gene expression by oligonol alleviated muscle loss by blocking nuclear translocation of NF-κB and FoxO3a in diabetic *db/db* mice. Oligonol normalized palmitate-induced senescent phenotype and aberrant cell cycle regulation and improved the impaired myotube formation in C2C12 muscle cells. Taken together, these results suggest oligonol as a supplement for alleviating muscle loss by suppressing atrogin-1 and MuRF1 expressions in diabetic mice and improving the impaired myotube formation via cell anti-senescence and cycle modulation in muscle cells.

## Figures and Tables

**Table 1:** Primer sequences.

Gene	Forward (5′–3′)	Reverse (5′–3′)
*MuRF1(Trim63)*	GTGTGAGGTGCCTACTTGCTC	GCTCAGTCTTCTGTCCTTGGA
*Atrogin-1(Fbxo32)*	CAGCTTCGTGAGCGACCTC	GGCAGTCGAGAAGTCCAGTC
*Ppara*	AACATCGAGTGTCGAATATGTGG	CCGAATAGTTCGCCGAAAGAA
*18s* rRNA	GGGAGCCTGAGAAACGGC	GGGTCGGGAGTGGGTAATTT

## Abbreviations

AMPKα	AMP-activated protein kinase α
FoxO3a	Forkhead box O3
MAFbx	Muscle atrophy f-box/atrogin-1
MuRF1	Muscle RING-finger protein 1
NF-κB	Nuclear factor-κB
OLG	Oligonol
ORO	Oil red O
PA	Palmitate
PPARα	Peroxisome proliferator-activated receptor α
SIRT1	Sirtuin1
